# Pharmacological Inhibition of Glutaminase 1 Normalized the Metabolic State and CD4+ T Cell Response in Sjogren's Syndrome

**DOI:** 10.1155/2022/3210200

**Published:** 2022-02-15

**Authors:** Jiayao Fu, Yiping Pu, Baoli Wang, Hui Li, Xiujuan Yang, Lisong Xie, Huan Shi, Zhijun Wang, Junhao Yin, Tianle Zhan, Yanxiong Shao, Changyu Chen, Qi Luo, Jiabao Xu, Zirui Zong, Xindi Wei, Wanwen Xiao, Chuangqi Yu, Lingyan Zheng

**Affiliations:** ^1^Department of Oral Surgery, Shanghai Ninth People's Hospital, College of Stomatology, Shanghai Jiao Tong University School of Medicine, Shanghai, China; ^2^National Center for Stomatology & National Clinical Research Center of Oral Disease, Shanghai, China; ^3^Shanghai Key Laboratory of Stomatology & Shanghai Research Institute of Stomatology, Shanghai, China; ^4^Laboratory of Oral Microbiota and Systematic Diseases, Shanghai Ninth People's Hospital, College of Stomatology, Shanghai Jiao Tong University School of Medicine, Shanghai, China; ^5^College of Stomatology, Shanghai Jiao Tong University, Shanghai, China

## Abstract

Previous studies have shown that abnormal metabolic reprogramming in CD4+ T cells could explain the occurrence of several autoimmune disorders, including Sjogren's syndrome (SS). However, therapeutic targets of the abnormal metabolism of CD4+ T cells remain to be explored. Here, we report that glutaminase 1 (Gls1), a pivotal factor in glutaminolysis, might be involved in the pathogenesis of SS. The expression of Gls1 was upregulated in infiltrated labial CD4+ T cells and circulating CD4+ T cells of SS patients. Inhibiting Gls1 with BPTES significantly abolished the proliferation rate, as indicated by EdU, CFSE, and Western blot analyses. Additionally, BPTES downregulated the extracellular acidification rate (ECAR) and oxygen consumption rate (OCR) values of activated CD4+ T cells from SS mice. *In vivo*, we injected different doses of BPTES into SS-like NOD/Ltj mice and found that 10 mg/kg BPTES significantly restored the salivary flow rate. Histological and qRT–PCR analyses showed that this concentration of BPTES attenuated lymphocytic infiltration and the numbers of PCNA-positive cells and CD4+ T cells. The proportions of IFN*γ*-producing cells and IL-17A-producing cells and the expression of several proinflammatory cytokines, including IFN*γ* and IL-17A, were also affected in the salivary glands of SS-like mice. Cytokine production in circulating serum was analyzed and showed that BPTES downregulated the effector functions of Th17 cells and Th1 cells. Collectively, these results indicate a positive relationship between Gls1 and SS development. Pharmacological inhibition of Gls1 with BPTES could normalize the effector functions of CD4+ T cells and effectively attenuate the symptoms of SS.

## 1. Introduction

Sjogren syndrome (SS) is an autoimmune disorder that primarily affects the exocrine glands, including the parotid glands, labial glands, and lacrimal glands [1]. The occurrence of SS is relatively high compared to that of other autoimmune disorders (approximately 0.5%-1% of the population), and approximately 1/3 of SS patients will develop rheumatoid arthritis (RA). Approximately 10% of SS patients will develop mild or severe systemic lupus erythematosus (SLE) or other autoimmune diseases [2–4]. Most investigators have noted the cooccurrence of these classic autoimmune disorders, as they share a similar pathogenetic process: the overactivation and hyperproliferation of CD4+ T cells [5]. For example, the pathogenesis of SS is characterized by CD4+ T cell infiltration in salivary glands. It was noted that CD4+ T cells were the majority (>75%) of infiltrated lymphocytes [5]. Additionally, an abnormal proportion of effector T cells (such as Th1 and Th17 cells) in the circulating blood is always observed in both SS and other autoimmune patients [6].

CD4+ T cell activation primarily includes cell cycle entry of naive T cells and distinct effector T cell subsets [7]. These processes require massive amounts of energy to support the proliferation and function of effector T cells. To fulfill energy demands upon activation, T cells undergo metabolic reprogramming to orchestrate the expression of hundreds of metabolic enzymes [8]. Therefore, major energy sources, including glucose, glutamine, and lipids, can be quickly used by cells. Although most mammalian cell types share similar metabolic processes to full the energy demands of each, some important metabolic pathways, including glucose metabolism, lipid metabolism, and amino acid metabolism, have always been mentioned and aroused more concern, as they selectively confer energy production upon activation, migration, and effector function of CD4+ T cells [9]. For example, upon activation, CD4+ T cells selectively elevate glycolysis, a process of glucose metabolism that takes up glucose into cells and converts it into pyruvate to fulfill the massive energy demand as the priming stages. Additionally, glutaminolysis, a process of amino acid metabolism that converts glutamine, a commonly existing animo-acid product, into glutaminate and alpha ketoglutarate (*α*-KG), which then enters the tricarboxylic acid (TCA) cycle [8, 10], was also activated to support the proliferation of CD4+ T cells. Targeting these processes has been demonstrated to alter the progression of several autoimmune diseases, including SS [11, 12]. However, the metabolic targets that could effectively attenuate the abnormal metabolic state of CD4+ T cells and reverse the progression of SS are still unknown.

Glutaminase 1 (Gls1) is the first enzyme in the glutaminolysis pathway and converts glutamine into glutamate [13]. It has been demonstrated that Gls1 can promote the formation of Th17 cells and partly regulate the effector functions of Th1 cells *in vivo* and *in vitro* [14]. Abnormal Gls1 expression can result in several autoimmune diseases, including SLE, experimental autoimmune encephalomyelitis (EAE), and RA [15, 16]. These investigations suggest potential regulatory and therapeutic targets that antagonize the effect of Gls1 on the development of autoimmune diseases. Several high-throughput sequences of both SS patients and activated T cells were used in our previous study [17, 18], and we found that Gls1 was significantly upregulated in both sequencing datasets and could serve as a potential target to interfere with the abnormal metabolic state of CD4+ T cells upon the development of SS. Therefore, in this study, we explored the pharmacological efficacy and function of targeting Gls1 to treat SS.

In this study, we showed that Gls1 was upregulated in the CD4+ T cells of SS patients. Pharmacological inhibition of Gls1 with BPTES normalized the metabolic state and restrained the proliferation of CD4+ T cells *in vitro*. *In vivo* injection of BPTES attenuated the progression of SS and decreased the proportions and effector functions of CD4+ T cells in the infiltrated salivary gland tissues of SS-like NOD/Ltj mice. Our study provides novel evidence that connects the metabolic regulation of CD4+ T cells and associated autoimmune diseases.

## 2. Results

### 2.1. Gls1 Was Upregulated in the CD4+ T Cells of SS Patients

**Figure 1:**
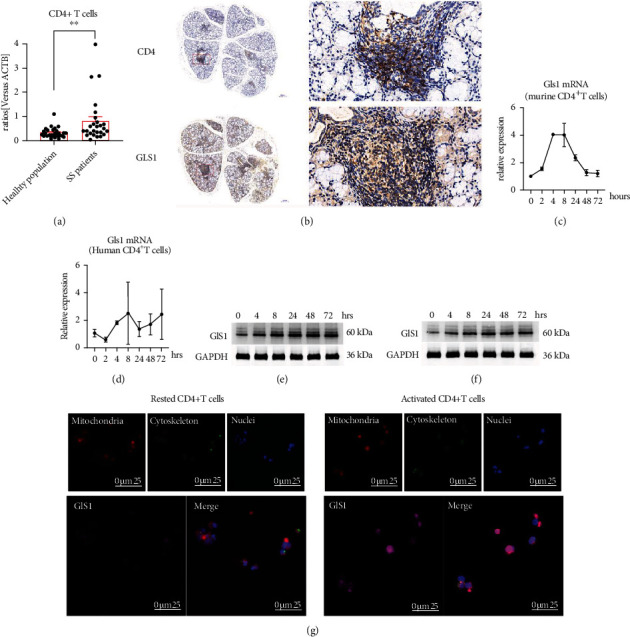
Gls1 was upregulated in SS patients and could be induced upon activation. (a) Expression of Gls1 in circulating CD4+ T cells obtained from healthy individuals and SS patients. *n* > 25. (b) Immunohistochemical staining of CD4-positive cells and Gls1-positive cells in the labial glands of SS patients. (c) qRT–PCR analysis of Gls1 mRNA expression in murine CD4+ T cells simulated with anti-CD3 and anti-CD28 antibodies in a time-dependent manner. (d) qRT–PCR analysis of Gls1 mRNA expression in human purified circulating CD4+ T cells simulated with anti-CD3 and anti-CD28 antibodies in a time-dependent manner. (e) Representative Western blot pictures of Gls1 protein expression in human purified circulating CD4+ T cells simulated with anti-CD3 and anti-CD28 antibodies. (f) Representative Western blot pictures of Gls1 protein expression in murine splenic CD4+ T cells simulated with anti-CD3 and anti-CD28 antibodies. (g) Representative picture of Gls1 expression and location in murine CD4+ T cells under a confocal microscope. Blue light indicates the position of nuclei; green light indicates the position of the cytoskeleton; red light indicates the position of mitochondria; violet light indicates the position and strength of Gls1. Summary data are presented as ^∗∗^*p* < 0.001 with unpaired two-tailed Student's *t*-tests.

To explore the metabolic signaling involved in the pathogenesis of SS, we combined two high-throughput sequences with the differentially expressed genes in infiltrated labial glands [17] and the differentially expressed genes in activated T cells [18] from our previous study. Of these, we noted that Gls1 was significantly upregulated. It has been shown that Gls1 is upregulated approximately 2-fold in labial glands compared with that in healthy individuals [17]. To further confirm this finding, we performed qRT–PCR and examined the gene expression of Gls1 in isolated lymphocytic subsets, especially CD4+ T cells. Consistent with previous data [17], we found that Gls1 was upregulated in CD4+ T cells isolated from SS patients by approximately 2-fold (Figure 1(a)).

We next used histological analysis to identify the expression and localization of Gls1 during SS development. Labial gland tissue was collected from SS patients. Samples were first validated by immunohistochemical analysis of CD4-positive cells (Figure 1(b) upper). The results showed that CD4+ T cells constituted the majority of infiltrating lymphocytes. Further immunohistochemical analysis showed that Gls1-positive cells (Figure 1(b) lower) were primarily present at these infiltrated sites.

Additionally, we stimulated purified human and murine CD4+ T cells *ex vitro* with anti-CD3 and anti-CD28 antibodies. The qRT–PCR results revealed that activation of both murine and human CD4+ T cells time-dependently induced Gls1 expression, especially at 4-8 hrs (Figures 1(c) and 1(d)). Consistent with the qRT–PCR results, the Western blot data indicated that Gls1 was elevated (Figures 1(e) and 1(f)). Interestingly, we found that the protein expression of Gls1 was stably upregulated at different time points, including 24-72 hrs after activation. We hypothesize that the protein stability of Gls1 may be changed upon CD4+ T cell activation. Previous studies have shown that upregulated Gls1 is always located in mitochondria, thereby supporting the proper function of the TCA cycle and oxidative phosphorylation (OXPHOS). We next identified whether the upregulation of Gls1 was associated with the localization of mitochondria in CD4+ T cells. As shown in Figure 1(g), Gls1 exhibited basal expression in the cytoplasm of resting CD4+ T cells. However, upon activation, the fluorescent strength of Gls1 was significantly upregulated, and a large proportion of their expression and localization were coincident with mitochondria in CD4+ T cells.

Collectively, these data show that Gls1 was abnormally upregulated in the CD4+ T cells of SS patients and might be involved in the pathogenesis of SS.

### 2.2. Inhibition of Gls1 by BPTES Ameliorated Glycolysis and OXPHOS in SS-Like CD4+ T Cells

**Figure 2:**
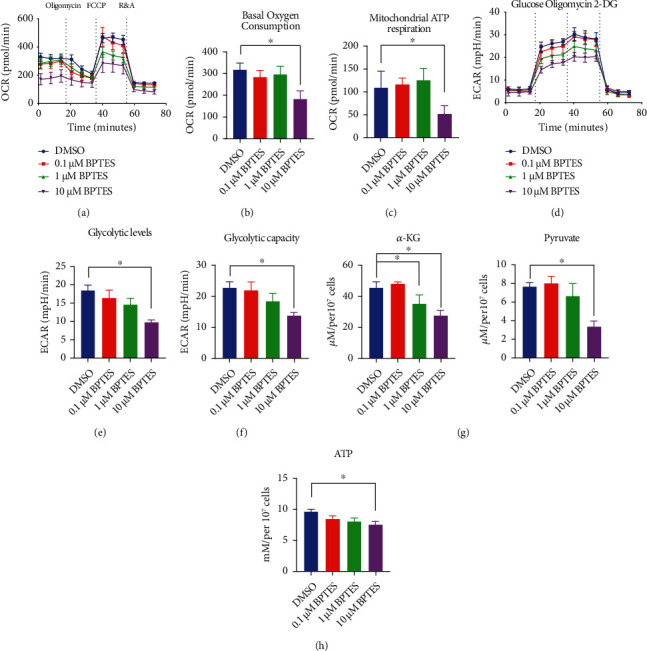
BPETS treatment normalized both glycolysis and OXPHOS in CD4+ T cells of SS-like NOD/Ltj mice. (a) The real-time OCR values in CD4+ T cells treated with 0.1 *μ*M to 10 *μ*M BPTES. (b) Measurement of basal oxygen consumption in CD4+ T cells treated with 0.1 *μ*M to 10 *μ*M BPTES. (c) Measurement of mitochondrial ATP respiration in CD4+ T cells treated with 0.1 *μ*M to 10 *μ*M BPTES. (d) The real-time ECAR values in CD4+ T cells treated with 0.1 *μ*M to 10 *μ*M BPTES. (e) Measurement of the basal glycolytic levels in CD4+ T cells treated with 0.1 *μ*M to 10 *μ*M BPTES. (f) Measurement of glycolytic capacity under stress in CD4+ T cells treated with 0.1 *μ*M to 10 *μ*M BPTES. (g) Measurement of *α*-KG and pyruvate concentration in CD4+ T cells treated with 0.1 *μ*M to 10 *μ*M BPTES. (h) Measurement of ATP concentration in CD4+ T cells treated with 0.1 *μ*M to 10 *μ*M BPTES. Summary data are presented as ^∗^*p* < 0.05 with unpaired two-tailed Student's *t*-tests.

Previous studies have shown that Gls1 supports the metabolic state of CD4+ T cells, which includes glutaminolysis and the potential regulation of glycolysis [16]. To identify the relationship between Gls1 and SS, we isolated splenic CD4+ T cells from SS-like model mice (NOD/Ltj mice) and examined the metabolic state of the cells with a Seahorse real-time metabolic analyzer. In our previous study, we found that the metabolic state was increased compared with that of wild-type ICR mice [11]. As shown in Figures 2(a)–2(c), Gls1 inhibition by BPTES downregulated the global OCR values, including basal oxygen consumption and mitochondrial-related ATP respiration, in a dose-dependent manner, especially at a concentration of 10 *μ*M (Figures 2(a)–2(c)).

We next examined the ECAR values, which indicate the levels of glycolysis, in CD4+ T cells treated with different doses of BPTES. As shown in Figures 2(d)–2(f), BPTES dose dependently downregulated the ECAR values of CD4+ T cells, indicating the downregulation of global glycolytic levels in response to extracellular glucose and oligomycin-induced glycolytic capacity. These results indicate that pharmacological inhibition of Gls1 by BPTES attenuated both glycolysis and OXPHOS *in vitro*, especially at a concentration of 10 *μ*M.

To further confirm that BPTES could influence the metabolic transformation of glucose and glutamine in CD4+ T cells, we examined the levels of intracellular pyruvate, *α*-KG, and ATP in the BPTES-treated groups. These metabolites primarily indicate the conversion products of glycolysis, glutaminolysis, and energy generation in cells. Our data showed that both *α*-KG and pyruvate levels were downregulated in cells treated with 1 *μ*M and 10 *μ*M BPTES (Figure 2(g)). ATP production showed that energy generation in CD4+ T cells was dose dependently affected by BPTES (Figure 2(h)).

Therefore, we concluded that BPTES-mediated inhibition could inhibit both glycolysis and OXPHOS in activated CD4+ T cells from SS model mice.

### 2.3. Inhibition of Gls1 by BPTES Ameliorated Glycolysis and OXPHOS in SS-Like CD4+ T Cells

**Figure 3:**
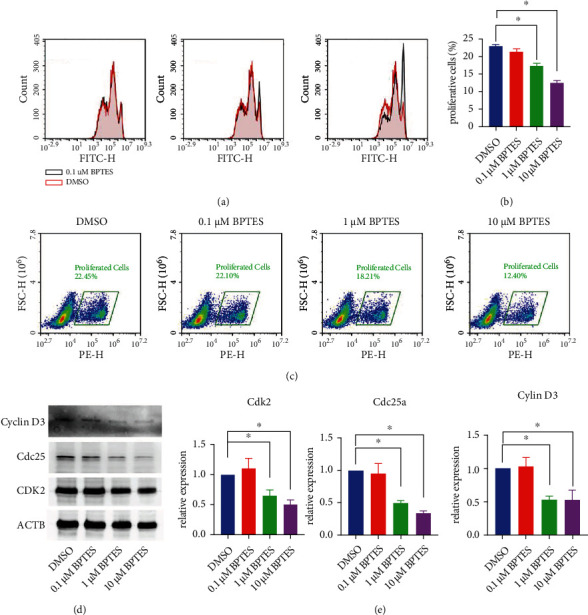
BPTES treatment confined the proliferation of CD4+ T cells upon activation. (a) Representative flow cytometry images of the CFSE assay in activated CD4+ T cells treated with the indicated doses of BPTES. (b, c) Representative flow cytometry images and statistical analysis of the EdU assay in activated CD4+ T cells treated with different dosages of BPTES. (d) Representative Western blot pictures of Cyclin D3, Cdc25, and Cdk2 in activated CD4+ T cells treated with different doses of BPTES. (e) ImageJ quantification of the relative expression of Cyclin D3, Cdc25, and Cdk2 in activated CD4+ T cells treated with different doses of BPTES. Summary data are presented as ^∗^*p* < 0.05 with unpaired two-tailed Student's *t*-tests.

Glycolysis and OXPHOS are the primary sources of energy in CD4+ T cells, supporting the quick expansion of CD4+ T cells upon activation. As the first enzyme involved in both glutaminolysis and glycolysis in CD4+ T cells, Gls1 has been shown to affect the proliferation and effector functions of CD4+ T cells, especially Th17 polarization. To identify whether Gls1 inhibition could attenuate the proliferation of CD4+ T cells, we used CFSE assays to investigate the proliferation levels of primary CD4+ T cells from NOD/Ltj mice. As shown in Figure 3(a), BPTES dose dependently interfered with the proliferation of CD4+ T cells, as indicated by increased proportions of nonproliferating cells. To further confirm these findings, we also examined proliferating cells by EdU staining. Similar to the CFSE results, treatment with BPTES dose dependently downregulated the numbers of proliferating cells, especially at concentrations of 1 *μ*M and 10 *μ*M (Figures 3(b) and 3(c)). We also determined the expression of several proliferation- and cell cycle-related proteins in activated CD4+ T cells in response to different doses of BPTES. The immunoblot data showed that pivotal indicators of CD4+ T cell proliferation, including PCNA, Cdc25a, and Cdk2, were significantly downregulated in response to 1-10 *μ*M BPTES (Figures 3(d) and 3(e)).

We next look forward to investigating the role of Gls1 in the potential migration of CD4+ T cells, as energy metabolic processes and products, such as glycolysis and lactate, have also been suggested to contribute to the alteration of migration capacity [19]. Traditionally, the migration and homing of CD4+ T cells into tissue are always accompanied by the downregulation of CD62L and CCR7 and the selective upregulation of homing molecules that target different nonlymphoid tissues, such as *α*4*β*7 integrin, CCR9, CCR4, and CCR10 [20]. Hence, we detected the expression of these genes in response to different dozes of BPTES upon CD4+ T cell activation. As indicated by qRT–PCR assays, we found that the expression of migration markers, including CD62L, CCR4, CCR7, CCR9, and CCR10, was not significantly changed (Supplemental Figure 1A). Additionally, flow cytometry detection of CD4+CD62L- T cell proportions was not changed in the BTPES-treated groups (Supplemental Figure 1B). Therefore, *ex vitro* assays indicated that inhibition of Gls1 by BPTES might be indispensable for the migration capacity of CD4+ T cells.

Collectively, we determined that pharmacological inhibition of Gls1 by BPTES restrained the proliferation of CD4+ T cells from SS model mice. Consistent with the findings of other studies that focused on the effect of Gls1 on the functions of CD4+ T cells, we believe that targeting Gls1 could efficiently slow the abnormal activation of CD4+ T cells.

### 2.4. BPTES Attenuated the Progression of SS and Decreased the CD4+ T Cell Population In Vivo

**Figure 4:**
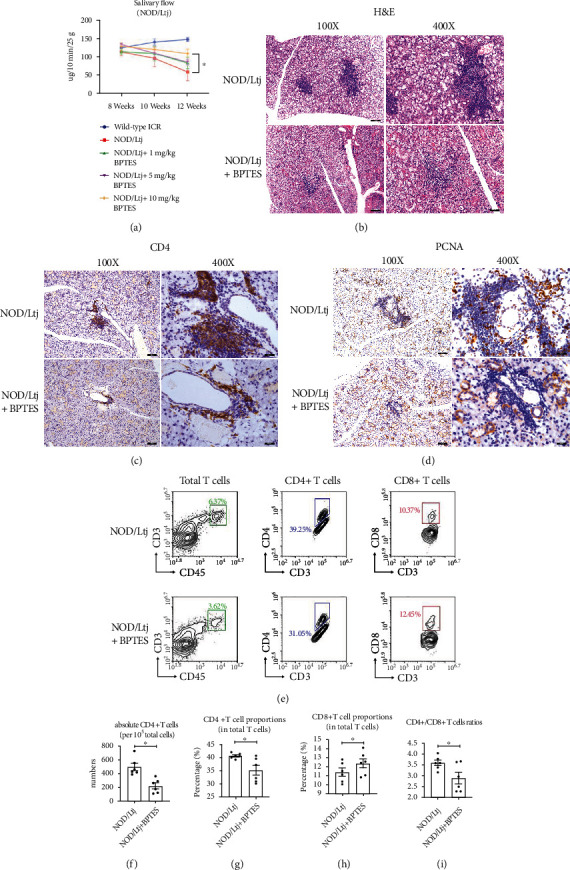
Treatment with BPTES alleviated the symptoms of SS and CD4+ T cell proportions in NOD/Ltj mice. (a) Simulated salivary flow rate of NOD/Ltj mice and wild-type ICR mice treated with the indicated doses of BPTES. The salivary flow rate was measured at the ages of 8 weeks, 10 weeks, and 12 weeks. (b) Pictures of H&E staining in NOD/Ltj mice treated with/without 10 mg/kg BPTES. (c) Pictures of immunohistochemical staining of CD4 in NOD/Ltj mice treated with/without 10 mg/kg BPTES. (d) Pictures of immunohistochemical staining of PCNA in NOD/Ltj mice treated with/without BPTES. (e) Representative picture of flow cytometry in salivary glands of the indicated NOD/Ltj mice. The proportions of CD4+ T cells (CD3+CD4+CD45+) and CD8+ T cells (CD3+CD8+CD45+) were gated and evaluated. (f) Measurement of the absolute CD4+ T cell numbers in NOD/Ltj mice treated with/without BPTES. (g) Measurement of the percentage of CD4+ T cells in total T cell populations in NOD/Ltj mice treated with/without BPTES. (h) Measurement of the percentage of CD8+ T cells in total T cell populations in NOD/Ltj mice treated with/without BPTES. (i) Measurement of CD4+/CD8+ T cell ratios in NOD/Ltj mice treated with/without BPTES. Summary data are presented as ^∗^*p* < 0.05 with unpaired two-tailed Student's *t*-tests.

We next aimed to determine the therapeutic potential of BPTES for the treatment of SS. We first determined salivary flow, which is a pivotal clinical indicator of SS, in NOD/Ltj mice in response to different doses of BPTES. The mice were subjected to 1 mg/kg, 5 mg/kg, and 10 mg/kg BPTES beginning at the occurrence of salivary deterioration (8 weeks), and the treatment was administered by intraperitoneal injection every 3 days. Two weeks and 4 weeks after injection (at the ages of 10 weeks and 12 weeks, respectively), the simulated salivary rate was determined in each group. As shown in Figure 4(a), at the age of 12 weeks, compared to wild-type ICR mice, NOD/Ltj mice exhibited significant defects in saliva secretion. Treatment with BPTES partly restored the salivary rate, indicating a potential role of BPTES in protecting mice from salivary dysfunction. Of note, treatment with 10 mg/kg BPTES mostly restored the salivary flow rate (Figure 4(a)). Therefore, we evaluated the histological differences in NOD/Ltj mice treated with or without BPTES. H&E staining showed that BPTES significantly downregulated the infiltration of lymphocytes in the salivary glands of NOD/Ltj mice (Figure 4(b)). We further evaluated the infiltration and proliferation of CD4+ T cells *in vivo* by immunohistochemistry. As shown in Figures 4(c) and 4(d), in the BPTES-treated groups, the numbers of CD4-positive cells was significantly decreased, and the numbers of PCNA-positive cells in infiltrating sites was decreased.

To further confirm that BPTES inhibited CD4+ T cell infiltration in NOD/Ltj mice, we digested and isolated single cell populations from the salivary glands of both groups. CD4+ T cells (CD45+CD3+CD4+CD8-) and CD8+ T cells (CD45+CD3+CD4-CD8+) were fluorescently labeled (Figure 4(e)). Flow cytometry showed that the absolute number of CD4+ T cells in the salivary gland was significantly decreased by BPTES (Figure 4(f)). Additionally, the proportion of CD4+ T cells in the total T cell population was also downregulated (Figure 4(g)). In comparison, the proportion of CD8+ T cells in the total T cell population was slightly upregulated (Figure 4(h)). Additionally, the ratios of CD4+ T cells to CD8+ T cells decreased in the BPTES-treated groups (Figure 4(i)), although CD4+ T cells were still the major T cell population in the salivary glands. Collectively, these results indicated that BPTES decreased the numbers and proportions of CD4+ T cells in infiltrated gland tissues.

### 2.5. BPTES Inhibited the Effector Functions of CD4+ T Cells upon SS Development

**Figure 5:**
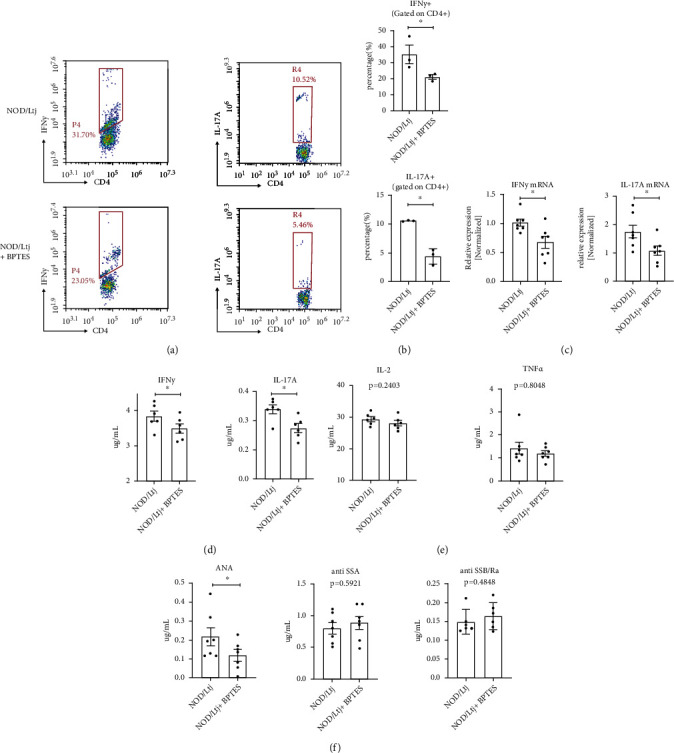
BPTES treatment selectively inhibited the effector function of CD4+ T cells and the immune response in NOD/Ltj mice. (a, b) Representative pictures and statistical analysis of flow cytometry data of IFN*γ*-producing and IL-17A-producing cells in the CD4+ T cell population. (c) qRT–PCR analysis of IFN*γ* and IL-17A gene expression in NOD/Ltj mice treated with/without 10 mg/kg BPTES. (d) ELISA quantification of IFN*γ* and IL-17A levels in circulating sera of the indicated NOD/Ltj mice. (e) ELISA quantification of IL-2 and TNF-*α* levels in circulating sera of the indicated NOD/Ltj mice. (f) ELISA quantification of ANA, anti-SSA, and anti-SSB levels in circulating sera of the indicated NOD/Ltj mice. Summary data are presented as ^∗^*p* < 0.05 with unpaired two-tailed Student's *t*-tests.

We next aimed to determine the potential regulatory effect of BPTES on the effector functions of CD4+ T cells in salivary glands. Previous studies have suggested that Gls1 inhibition promotes both Th1- and Th17-based differentiation *in vivo* and *in vitro*. We therefore hypothesized that treatment with BPTES might also interfere with the numbers of Th1 or Th17 cells among tissue-infiltrated CD4+ T cells. To confirm this hypothesis, we first used flow cytometry to further identify the components of infiltrated CD4+ T cells in the salivary glands of NOD/Ltj mice. As shown in Figures 5(a) and 5(b), treatment with BPTES decreased the proportions of IFN*γ*-producing cells among CD4+ T cells to approximately 70%, whereas the proportions of IL-17A-producing cells among total CD4+ T cells decreased to approximately 50%. We further measured the mRNA levels of IFN*γ* and IL-17A in the salivary glands of the mice, and the qRT–PCR results showed that the expression of both IFN*γ* and IL-17A was downregulated in NOD/Ltj mice treated with BPTES (Figure 5(c)). Finally, we measured the serum IFN*γ* and IL-17A levels by ELISA, and the results showed that the serum concentrations of both IFN*γ* and IL-17A were decreased in BPTES-treated NOD/Ltj mice. In addition to these classical proinflammatory cytokines, several cytokines related to Th1 cells were slightly but not significantly downregulated, including IL-2 and TNF-alpha (Figure 5(d)). Collectively, these results indicate that treatment with BPTES partially inhibited the effector functions of CD4+ T cells upon SS development.

Interestingly, when we measured the humoral autoantigen levels, including those of anti-SSA, anti-SSB, and ANA, in NOD/Ltj mice, we found that at 12 weeks old (approximately 4 weeks after BPTES injection), BPTES failed to downregulate the levels of autoantigens related to SS development, whereas the serum levels of ANA were downregulated. These results might be because BPTES primarily inhibits the immune response of T cells but has less effect on the humoral immune response of B cells (Figure 5(e)).

## 3. Materials and Methods

### 3.1. Animals

Female NOD/Ltj mice, ICR mice, and C57/BL6 mice were provided by the Model Animal Research Center of Nanjing University (China). The disease progression of female NOD/Ltj mice was assessed in our previous study [11]. At the age of 8 weeks, mice in the different groups were treated intraperitoneally with different drugs. BPTES (S7753, Selleck, US) was dissolved in 2% DMSO (D8418, Sigma–Aldrich, US), 2% Tween-80 (P5188, Sigma–Aldrich, US), 30% PEG2000, and 66% ddH_2_O. Mice in the BPTES groups (1 mg/kg, 5 mg/kg, and 10 mg/kg) were intraperitoneally administered the treatments every 3 days for 4 weeks. Mice in the control group were administered 0.1 ml of the negative control solution (2% DMSO, 2% Tween-80, 30% PEG2000, and 66% ddH_2_O). At the age of 12 weeks, the mice were anesthetized and underwent saliva flow rate detection, blood collection, and salivary gland collection; afterward, the mice were sacrificed. During the experiment and routine feeding, the animals received humane care according to the criteria outlined in the Guide for the Care and Use of Laboratory Animals published by the National Institutes of Health (NIH). The study was conducted according to the guidelines of the Declaration of Helsinki and approved by the Ethics Committee of the Shanghai Ninth People's Hospital affiliated to Shanghai Jiao Tong University School of Medicine (Approval sequence: SH9H-2021-TK69-1).

### 3.2. Cell Culture and Isolation

Primary CD4+ T cells were isolated from the lymph nodes and spleens of 8-week-old NOD/Ltj mice or C57/BL6 mice with a CD4+ T cell isolation kit (18952, StemCell Technology, Canada) according to the experimental design. CD4+ T cells were cultured in RPMI 1640 medium (11875093, Gibco, US) supplemented with 10% fetal bovine serum (10099141, Gibco, US) and 1% penicillin/streptomycin (15070063, Gibco, US). CD4+ T cells were activated with T-activator CD3/CD28 microbeads (11452D, Gibco, US) according to the manufacturer's protocol. Unless otherwise noted, cells were pretreated with the indicated doses of BPTES and then stimulated with CD3/CD28 microbeads for 24 hrs.

### 3.3. Biological Sample Isolation

Blood samples from SS patients (*n* > 30) and healthy donors and labial gland samples from SS patients were collected from the Department of Oral Surgery of Shanghai Ninth People's Hospital affiliated with Shanghai Jiao Tong University School of Medicine. Peripheral blood mononuclear cells (PBMCs) were separated by gradient centrifugation with Ficoll-Paque (17-5442-02, GE Healthcare, US) according to the manufacturer's instructions. Human CD4+, CD8+, and CD19+ cells were isolated with the corresponding microbeads (130-090-877/130-090-878/130-090-880, Miltenyi Biotec, Germany). All sample donors provided written informed consent. The study was approved by the Ethics Committee of Shanghai Ninth People's Hospital affiliated with Shanghai Jiao Tong University School of Medicine (No. SH9H-2019-T159-2).

### 3.4. Real-Time Metabolic Extracellular Acidification Rate (ECAR) and Oxygen Consumption Rate (OCR) Analysis

The ECAR and cellular OCR of CD4+ T cells were determined by a Seahorse XFe96 analyzer (XFe96, Agilent, US). CD4+ T cells (2 × 10^5^ cells per well) were cultured in Seahorse 96-well culture plates that were precoated with Cell-Tak (DLW354240, Corning, US). The ECAR and OCR were measured under basal conditions and in response to three doses of BPTES. Briefly, CD4+ T cells were treated as indication. Prior to detection, cells were plated on a Seahorse 96-well culture plate, which was precoated with Cell-Tak for adhesion.

### 3.5. Quantitative Real-Time PCR (qRT–PCR)

Cells were lysed with TRIzol reagent (9108Q, Takara, Japan). RNA was extracted and reverse transcribed (RR036A, Takara, Japan) in preparation for qRT–PCR analysis (RR420A, Takara, Japan). Primer sequences are listed in Table 1.

### 3.6. Western Blots

For the Western blot analysis, at least 1∗10^7^ CD4+ T cells in each group were collected by centrifugation. Cells were washed with ice-cold PBS 3 times prior to ultrasonic lysis. The following antibodies were purchased from Invitrogen and Cell Signaling Technology (CST): *β*-actin (#4970), GAPDH (#2118), Cdc25a (#3652), Cdk2 (#18048), and Cyclin D3 (#2936). Gls1 antibody was purchased from Invitrogen (PA5-35365) and Cell Signaling Technology (#56750). The membranes were incubated with the indicated primary antibodies overnight at 4°C at a 1 : 1000 dilution and secondary antibodies (G-21234, Invitrogen) for 1.5 h at 37°C at a 1 : 1000 dilution. Images were captured with an Amersham Imager 600.

### 3.7. Histological Analysis

Hematoxylin and eosin (H&E) staining was described in our previous study. For immunohistochemical staining, deparaffinized sections underwent antigen retrieval, antigen blocking, overnight primary antibody incubation at 4°C (CD4 (25229, Cell Signaling Technology, US)), and PCNA (13110, Cell Signaling Technology, US) ant at a 1 : 500 dilution and DAB staining (8801-4965-72, eBioscience, US).

### 3.8. Simulated Salivary Flow Rate Evaluation

Two and four weeks after BPTES injection (at the ages of 10 weeks and 12 weeks, respectively), the simulated salivary rate in each group was determined. The process of salivary flow determination and the methods of normalization were described in our previous study [11]. Briefly, mice were anesthetized with sodium pentobarbital and injected with 0.5 mg/kg pilocarpine hereafter. Five minutes after pilocarpine injection, saliva was collected by a dry cotton ball for 10 minutes. Of note, if the dry cotton ball was filled with saliva, another preweighed dry cotton ball was replaced to ensure that all the saliva was collected. Collected saliva was normalized to the weight (25 g) of the indicated mice.

### 3.9. Enzyme-Linked Immunosorbent Assay (ELISA)

Serum of NOD/Ltj mice was collected and separated from heart blood for cytokine analysis. The following kits were used in this study: mouse IFN*γ* Quantikine ELISA kit (MIF00, R&D Systems, US) and mouse IL-17 Quantikine ELISA kit (M1700, R&D Systems, US). The procedure was performed according to the manufacturer's instructions.

### 3.10. Flow Cytometry-Based Proliferation Assays

5-(and-6)-Carboxyfluorescein diacetate succinimidyl ester (CFSE) and 5-ethynyl-2-deoxyuridine (EdU) kits were purchased from Thermo Fisher (C34554, Thermo Fisher, US) and Beyotime (C0075S, China). CFSE and EdU labeling was performed according to our previous study and the manufacturer's protocol [11]. The fluorescence intensity was measured by an Agilent flow cytometer.

### 3.11. Statistical Analysis

Statistical analyses were performed using the GraphPad Prism 8.0 software. Unless indicated otherwise, the evaluated parameters in the groups are shown as the means and standard deviations of the mean (SEM) for each group. All results were compared with 2-tailed *t*-tests.

## 4. Discussion

In this study, we first showed that Gls1 was abnormally upregulated in the CD4+ T cells of SS patients. Further *in vivo* and *in vitro* analysis showed that pharmacological inhibition of Gls1 by BPTES decreased the proliferation and effector functions of CD4+ T cells. Additionally, the progression of SS was significantly delayed by the administration of BPTES. Mechanistically, similar to a previous report, the inhibition of Gls1 by BPTES normalized both glycolysis and OXPHOS in CD4+ T cells from SS model mice. These data reveal a potential regulatory effect of Gls1 on CD4+ T cell overactivation and the development of SS.

Targeting the dysfunctional metabolism of CD4+ T cells to treat autoimmune disease has aroused widespread concern in recent decades. The principle of targeting CD4+ T cell metabolism is important because the metabolic state not only supports cell survival but also selectively supports effector functions in different T cell subsets. As previously described, there are three major kinds of nutrients that are required for the proper functioning of CD4+ T cells: glucose, amino acids, and lipids. The digestion of glucose primarily involves both glycolysis and oxidative phosphorylation. Glycolysis takes place in the cytoplasm and converts glucose to pyruvate, which is further converted to either lactate or acetyl coenzyme A (acetyl-CoA) in the mitochondria. Mitochondrial acetyl-CoA subsequently enters the TCA cycle through oxidative phosphorylation. An increase in glycolysis always occurs in hypoxic microenvironments under normal conditions. However, upon CD4+ T cell activation or tumor cell proliferation, cells prefer the glycolysis-lactate production pathway even in the presence of oxygen. These unique changes are also known as aerobic glycolysis or the Warburg effects in immune or cancer cells [20]. Compared with the effect on quiescent cells, elevated glycolysis supported the functions of Th1 and Th17 cells and Tfh cells. For example, the gatekeeper of glucose uptake, Glut1, is essential for glucose utilization, reducing T cell proliferation and differentiation to effector T cells associated with autoimmunity [21]. Pyruvate kinase M2 (PKM2), a kinase that converts lactate into pyruvate, has a distinctive role in the effector function of CD4+ T cells due to its dimeric/monomeric structure in the cytoplasm and tetrameric structure in the nucleus [22]. Other investigators also reported that some glycolytic kinases, such as PKM2, performed their functions independent of metabolic regulation. Damasceno et al. reported that PKM2 fine-tuned the activation of STAT3, thereby supporting the differentiation of Th17 cells in autoimmune EAE mouse models [23]. In addition to targeting the pivotal glycolysis kinase, many studies have focused on the transcription factors that activate the transcription of glycolysis and glutaminolysis genes HIF-1alpha and Myc [24]. For example, HIF-1alpha upregulation orchestrates the imbalance in Th17/Treg cells, thereby supporting the development of autoimmunity [25]. Myc deficiency also leads to a failure of metabolic reprogramming in CD4+ T cells, which impairs proliferation and cellular size *in vivo* and *in vitro* [8].

In addition to targeting glycolysis to treat autoimmunity, many studies have focused on the regulation of lipid metabolism, especially de novo fatty acid synthesis [26] and cholesterol biosynthesis [27]. These processes primarily produce energy and essential energy products through mitochondrial OXPHOS and have been proven to be essential for the differentiation of Th17 cells [28]. Compared to studies on regulating glucose or lipid metabolism in CD4+ T cells, many studies have focused on glutaminolysis, the biological pathway that degrades glutamine through OXPHOS, to interfere with the proliferation and effector functions of CD4+ T cells. In vitro, glutamine depletion could decrease CD4+ T cell proliferation and elevate apoptosis [14, 29]. The degradation and conversion of glutamine are also called glutaminolysis and involve the hydrolyzation of glutamine to glutamate, which is then metabolized to *α*-KG by several glutaminases [30]. The levels of glutaminolysis can also be modulated by OXPHOS in cells. Studies of autoimmunity have primarily focused on membrane glutamine transporters such as ACST2 [31] or glutaminases such as Gls1, which is the main topic in this study.

Metabolic regulation is a connected network that could be influenced by different pathways. This phenotype is associated with distinct metabolic changes when different molecular targets are altered. For example, inhibiting glutaminolysis could lead to a deficiency in amino acid products, thereby inhibiting both lipogenesis [32] and glycolysis [31] in cells. Hence, the goal of this research was to utilize the classic regulation of glutaminolysis, thereby modulating multiple metabolic processes, including glycolysis and OXPHOS, in CD4+ T cells. Gls1 is one of the most important kinases in glutaminolysis. Gls1 has been implicated in several autoimmune diseases, including SLE [19], EAE [19], and RA [15]. We proposed that targeting Gls1 could be an excellent therapeutic strategy in the treatment of autoimmune disorders. However, before this study, the function of Gls1 had not been verified in SS research.

Additionally, in this study, there were several interesting phenotypes. For example, we found that BPTES divergently inhibited IFN*γ*-producing cells in the infiltrated CD4+ T cell population of salivary gland tissues, as indicated by flow cytometry. Here, we supposed several possible reasons. The first is that Gls1 inhibition divergently inhibited the expression of IFN*γ* in CD4+ T cells. This assumption was based on the investigation of Johnson et al., who found that glutaminase inhibited Th1/CTL effector function through chromatin modification of alpha-KG. Therefore, the inhibition of IFN*γ* production was temporary or even elevated initially but exhausted over time [14]. This research partly demonstrated why there were several IFN*γ*+ subproportions in infiltrated CD4+ T cells. Another possible explanation is that BPTES inhibition epigenetically regulated the expression of IFN*γ* or its related genes, leading to distinct expression of IFN*γ* in Th1 cells. This can be indicated by several studies, which reported that Gls1/glutaminolysis is required for histone acetylation in CD4+ T cells [13]. Obviously, studies related to epigenetic regulation by Gls1 need to be further addressed in SS and other autoimmune diseases.

Among the known inhibitors of Gls1, BPTES is primarily used by researchers to treat inflammatory or immune-related diseases. Wang et al. reported that BPTES prevented the anthrax lethal toxin-induced inflammatory response in Balb/c mice [33]. Le et al. showed that BPTES inhibited the proliferation and survival of B cells by modulating the TCA cycle [34]. Xia et al. showed that BPTES downregulated the acetylation of histone H3 in the IL-17A promoter, thereby decreasing the production of IL-17A in psoriasis mouse models [13]. Consistent with previous studies of Gls1 or BPTES in autoimmune disorders, we suggest that further clinical trials or biological safety analyses are recommended for the drug design of BPTES as a Gls1 inhibitor.

## Figures and Tables

**Table 1:** Primer sequences of indicated genes.

Gene name (species)	Forward primer sequence (5′-3′)	Reverse primer sequence (5′-3′)
Glutaminase 1 (human)	AGGGTCTGTTACCTAGCTTGG	ACGTTCGCAATCCTGTAGATTT
*β*-Actin (human)	CATGTACGTTGCTATCCAGGC	CTCCTTAATGTCACGCACGAT
Glutaminase 1 (mouse)	TTCGCCCTCGGAGATCCTAC	CCAAGCTAGGTAACAGACCCT
IFN*γ* (mouse)	ATGAACGCTACACACTGCATC	CCATCCTTTTGCCAGTTCCTC
IL-17 (mouse)	TTTAACTCCCTTGGCGCAAAA	CTTTCCCTCCGCATTGACAC
*β*-Actin (mouse)	GGCTGTATTCCCCTCCATCG	CCAGTTGGTAACAATGCCATGT
CD62L (mouse)	TACATTGCCCAAAAGCCCTTAT	CATCGTTCCATTTCCCAGAGTC
CCR4 (mouse)	GGAAGGTATCAAGGCATTTGGG	GTACACGTCCGTCATGGACTT
CCR7 (mouse)	TGTACGAGTCGGTGTGCTTC	GGTAGGTATCCGTCATGGTCTTG
CCR9 (mouse)	CTTCAGCTATGACTCCACTGC	CAAGGTGCCCACAATGAACA
CCR10 (mouse)	GGACTTTACTCCGGGTACGAT	CAGGGAGACACTGGGTTGGA

## Data Availability

The datasets generated for this study can be found in the Figshare (doi:10.6084/m9.figshare.19091102).

## Supplementary Materials

Supplementary Materials Supplementary Figure 1: expression of T cell migration factors in response to different doses of BPTES.
