# Supplementary material for: Pharmacological Inhibition of Glutaminase 1 Normalized the Metabolic State and CD4+ T Cell Response in Sjogren's Syndrome
Source: J Immunol Res. 2022 Feb 15;2022:3210200. doi: 10.1155/2022/3210200 (PMC8863479; doi:10.1155/2022/3210200)
Supplement: Supplementary Materials — Supplementary Figure 1: expression of T cell migration factors in response to different doses of BPTES. [file 3210200.f1.pdf]

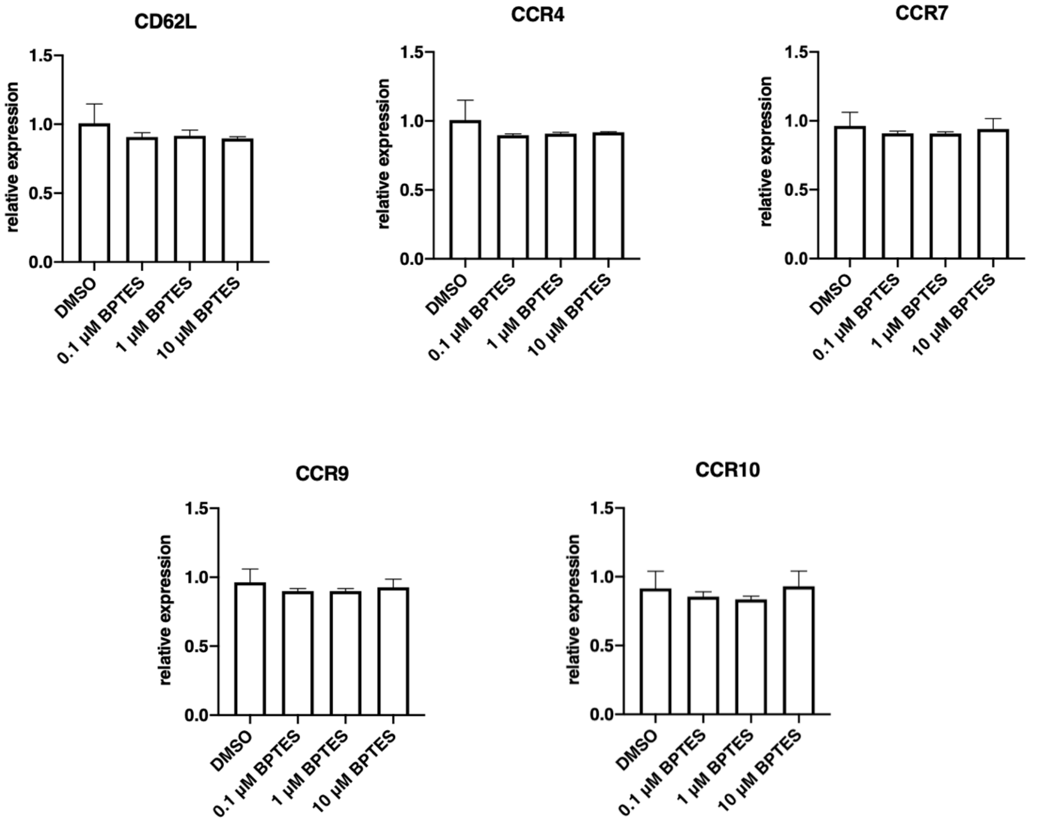

Supplemental Figure 1. Expression of T cell migration factors in response to different doses of BPTES.
